# Low-Dose CT Fluoroscopy-Guided Drainage of Deep Pelvic Fluid Collections after Colorectal Cancer Surgery: Technical Success, Clinical Outcome and Safety in 40 Patients

**DOI:** 10.3390/diagnostics13040711

**Published:** 2023-02-13

**Authors:** Robert Stahl, Max Seidensticker, Giovanna Negrão de Figueiredo, Vera Pedersen, Alexander Crispin, Robert Forbrig, Yigit Ozpeynirci, Thomas Liebig, Melvin D’Anastasi, Danilo Hackner, Christoph G. Trumm

**Affiliations:** 1 Institute for Diagnostic and Interventional Neuroradiology, University Hospital, Ludwig-Maximilians-University (LMU), Marchioninistr. 15, 81377 Munich, Germany; 2 Department of Radiology, University Hospital, Ludwig-Maximilians-University (LMU), Marchioninistr. 15, 81377 Munich, Germany; 3 Department of Neuroradiology, University Hospital Zurich, University of Zurich, Frauenklinikstr. 10, 8091 Zurich, Switzerland; 4 Department of Orthopaedics and Trauma Surgery, Musculoskeletal University Center Munich (MUM), University Hospital, Ludwig-Maximilians-University (LMU), Marchioninistr. 15, 81377 Munich, Germany; 5 IBE—Institute for Medical Information Processing, Biometry and Epidemiology, Ludwig-Maximilians-University (LMU), Marchioninistr. 15, 81377 Munich, Germany; 6 Medical Imaging Department, Mater Dei Hospital, University of Malta, MSD 2090 Msida, Malta; 7 Department of General and Visceral Surgery, University Hospital Erlangen, Friedrich-Alexander-University (FAU) Erlangen-Nuremberg, Krankenhausstr. 12, 91054 Erlangen, Germany

**Keywords:** technical outcome, clinical outcome, CT-guided drainage, colorectal surgery, pelvic fluid collection

## Abstract

Purpose: To assess the technical (TS) and clinical success (CS) of CT fluoroscopy-guided drainage (CTD) in patients with symptomatic deep pelvic fluid collections following colorectal surgery. Methods: A retrospective analysis (years 2005 to 2020) comprised 43 drain placements in 40 patients undergoing low-dose (10–20 mA tube current) quick-check CTD using a percutaneous transgluteal (*n* = 39) or transperineal (*n* = 1) access. TS was defined as sufficient drainage of the fluid collection by ≥50% and the absence of complications according to the Cardiovascular and Interventional Radiological Society of Europe (CIRSE). CS comprised the marked reduction of elevated laboratory inflammation parameters by ≥50% under minimally invasive combination therapy (i.v. broad-spectrum antibiotics, drainage) within 30 days after intervention and no surgical revision related to the intervention required. Results: TS was gained in 93.0%. CS was obtained in 83.3% for C-reactive Protein and in 78.6% for Leukocytes. In five patients (12.5%), a reoperation due to an unfavorable clinical outcome was necessary. Total dose length product (DLP) tended to be lower in the second half of the observation period (median: years 2013 to 2020: 544.0 mGy*cm vs. years 2005 to 2012: 735.5 mGy*cm) and was significantly lower for the CT fluoroscopy part (median: years 2013 to 2020: 47.0 mGy*cm vs. years 2005 to 2012: 85.0 mGy*cm). Conclusions: Given a minor proportion of patients requiring surgical revision due to anastomotic leakage, the CTD of deep pelvic fluid collections is safe and provides an excellent technical and clinical outcome. The reduction of radiation exposition over time can be achieved by both the ongoing development of CT technology and the increased level of interventional radiology (IR) expertise.

## 1. Introduction

Colorectal carcinoma is a common cancer worldwide, with 1.8 million reported cases in 2018. It caused 881,000 deaths, making the disease the second most common cause of cancer-related deaths [1]. The incidence varies depending on the criteria applied. For example, it is shown to be decreasing in western populations [2], while in the age group under 50 years an increase is observed [3]. Women are more frequently affected than men [4]. Surgery, radiation therapy and chemotherapy are the key components of rectal cancer therapy, while surgery is the only curative therapy for localized colorectal cancer. Adjuvant chemotherapy is usually recommended for patients with lymph node metastases [5].

Postoperatively, fluid collections of varying etiology may occur. These are usually seromas, hematomas or lymphoceles, which can become superinfected in a germ-rich environment despite perioperative antibiotic therapy [6]. In addition, in colorectal surgery, anastomotic leakage (AL) may occur. It is defined as ’peritonitis caused by leakage, pelvic abscess, or discharge of faeces from the pelvic drain, including leakage from any stapler line at any time postoperatively’ [7] and is still associated with significant morbidity and mortality [8]. In the postoperative period, AL may be verified at different time points, including so called early leakages (during initial hospital stay) and late leakages (after discharge from hospital) [7]. Available diagnostic tools for the verification of an AL are a CT scan, contrast enema, endoscopy, and reoperation [9]. Rahbari et al. [10] suggested a three-grade scale grading system for postoperative AL, comprising Grade A (no therapeutic intervention), Grade B (active intervention without laparotomy) and Grade C (laparotomy required).

Since its introduction and broad use during the 1980s, image-guided percutaneous drainage (PD) has become part of the standard therapeutic armamentarium in patients with symptomatic abdominal and pelvic fluid collections [11,12]. In comparison to sequential CT guidance [13], CT fluoroscopic guidance allows for near real time visualization of needle or drain insertion similar to ultrasound (US), even in cases of difficult access routes and uncooperative patients [14,15,16]. Minimally-invasive percutaneous drainage can either have a temporizing effect in order to stabilize the patients’ general condition before reoperation, or even a therapeutic effect, circumventing revision surgery [8,17]. The percutaneous transgluteal approach has been described as a safe and effective alternative to surgery, particularly in deep pelvic fluid collections [18,19,20,21]. If such access is not possible due to the interposition of critical structures (e.g., arteries, nerves), a transperineal access can be used [22,23]. The main alternatives are the percutaneous transabdominal anterior or lateral approach—guided by ultrasound (US) or CT [24]—and the endoscopic US-guided approach [25,26,27].

The aim of this retrospective study is to report the technical and clinical success of percutaneous pelvic drain placement guided by low milliampere quick-check CT fluoroscopy in a patient cohort presenting with symptomatic deep pelvic fluid collections after colorectal cancer surgery, without and with early as well as late AL, comprising Rahbari Grade B and C leakages.

## 2. Materials and Methods

### 2.1. Study Subjects

**Figure 1:**
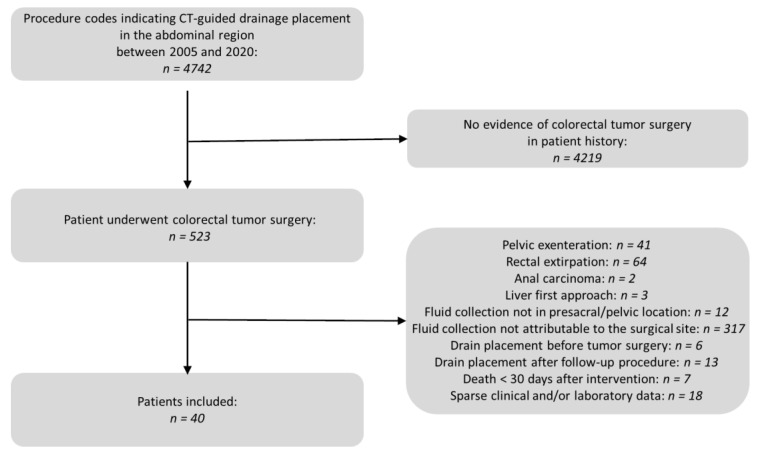
Flow chart of the patient selection process.

All patients between 2005 and 2020 with examination codes indicating CT-guided drain placement in the abdominal region were searched in the database of the Radiology Information System (RIS). From this, all patients with colorectal tumor surgery in the records were extracted. Exclusion criteria were as follows: (1) pelvic exenteration due to the lack of comparability in this case of major surgical corridor damage; (2) rectal extirpations, as these usually receive a definitive colostomy and thus no intestinal anastomosis in the actual sense is performed; (3) patients where drain placement occurred after surgery for anal carcinoma; (4) patients undergoing primary surgery of the liver as part of a “liver first approach” for synchronous hepatic metastatic carcinoma; (5) patients with fluid collections not in presacral/pelvic locations; (6) patients undergoing drain placement into a fluid collection that could not be assigned to the surgical site; (7) patients who had received the drain before surgery; (8) patients undergoing drain placement after a follow-up procedure after the actual tumor surgical operations (ileostomy or colostomy reversal in most cases); (9) patients who died within 30 days of drain placement and where a relationship between the intervention and cause of death could be ruled out; and (10) patients with insufficient clinical and/or laboratory data. An overview of the patient selection process is depicted in Figure 1.

The ethics committee of the Ludwig-Maximilians University of Munich (number 22-0030, 18 February 2022) had approved the study in advance. All interventions were performed according to the Declaration of Helsinki. Informed consent by adults or legal guardians for permission to undergo CTD was usually acquired at least 24 h or—in case of an emergency indication—directly prior to each procedure after detailed explanation of the planned therapeutic intervention.

### 2.2. CT Imaging Protocol

The indication for drain placement had been extensively discussed and verified by abdominal surgeons and interventional radiologists in a multidisciplinary team meeting. The clinical patient charts and radiological images from the individuals who had been transferred to our department for drain placement in symptomatic (i.e., pain, fever) pelvic fluid collections after colorectal tumor surgery were retrospectively investigated by two board certified interventional radiologists (IRs), each with more than 15 years of experience in CT-guided intervention.

Before the planned intervention, previous contrast-enhanced cross-sectional images (CT or MRI) not older than 48 h were thoroughly analyzed. All procedures were carried out on a 16-(Somatom Sensation 16, Siemens Healthineers, Erlangen, Germany) or 128-slice (Somatom Definition AS+; Somatom Definition Edge, Siemens, Munich, Germany) CT scanner equipped with fluoroscopy (CARE Vision CT^®^, Siemens Healthineers, Erlangen, Germany). An unenhanced CT scan of the abdomen in prone or lateral decubitus position was generally performed before and after each CTD placement to plan the access trajectory and rule out postinterventional adverse events. The pre-interventional CT scan was characterized by 5 mm slices and coronal and sagittal reconstructions. This CT scan was correlated to the diagnostic contrast-enhanced CT scan on which the indication for drainage was decided. With respect to radiation protection, thyroid shields, aprons, and eyeglasses of 0.5 mm lead equivalent were used by the IR. Before sterile draping, an additional shield was put onto the lower half of the patient to reduce scattered radiation. Angular beam modulation (Hand Care^®^) was activated during CTD in order to reduce radiation exposure of the operator’s hands, i.e., the radiation exposure is switched off between eleven and three o’clock positions of the X-ray tube. 

Patients with severe cardiorespiratory comorbidities were monitored by pulse oximetry periprocedurally. Local anesthesia with 10 to 20 mL of 2% Mepivacaine hydrochloride was applied after sterile draping and disinfection of the skin overlying the planned drain entry point. After a minimal skin incision, the drain (Flexima^TM^ All Purpose Drainage, Boston Scientific Corporation or ReSolve^®^ Non-Locking Drainage Catheter, Merit Medical, South Jordan, UT, USA) was inserted and advanced to the fluid collection under the use of the curved trocar-technique under intermittent quick-check CTF [15,28].

Following drain placement within the fluid collection, an unenhanced CT scan covering at least 10 cm above and below the entry point along the z-axis was performed in order to confirm the correct final drain position and rule out immediate complications. The drain was then fixed at the skin level with a suture and covered with a sterile bandage. All patients were monitored clinically for at least 24 h.

### 2.3. Analysis of Pre-, Peri- and Post-Interventional Period

Two experienced IRs (R.S.; C.G.T.) evaluated the technical and clinical outcome in a retrospective investigation of patients’ imaging studies available in the local PACS, radiology reports and remaining medical records, as well as the complications associated with CTF-guided drain placement during a post-interventional period of 30 days. The following variables were assessed: indications for colorectal surgery, surgical techniques, predominant locations of fluid collection, interventional techniques (Trocar vs. Seldinger technique), the number of drains, the diameter of drainage catheters, the access trajectory for drainage and peri-interventional complications according to the SIR criteria [29]. Measurements of the mean diameter of fluid collections were taken and fluid collection entities were differentiated. The angle and height of the drain insertion was determined as described in Supplementary Figure S1.

Technical success was described as drain insertion within the fluid collection with consecutive aspiration for microbiological analysis and volume reduction of the fluid collection by at least 50%. In the case that the drain could not be inserted into the fluid collection or could not be aspirated, a technical failure was noted [30].

Complications were documented as defined by the CIRSE classification [31].

Per definition, clinical success required a normalization or marked improvement of clinical symptoms and inflammatory parameters (C-reactive Protein, Leukocyte count, Interleukin-6) under minimally invasive combination therapy (intravenous broad-spectrum antibiotics, drainage) within one month after the intervention. Additionally, clinical success was defined by the absence of the need for any further surgical procedure related to the intervention. The clinical outcome was subsequently compared with the applied surgical techniques to detect possible causal relations.

CT dosimetry was performed according to Kloeckner et al. [32] for all interventions using the dose length product (DLP), documented by the CT scanner as primary dosimetric quantity data. DLP was analyzed for the pre-interventional planning CT scan, the sum of all intra-interventional CT fluoroscopic acquisitions, and the post-interventional control CT scan.

The microbiological results of the secretion delivered by the drainage catheters were evaluated. The removal dates of each patient’s drain were registered.

### 2.4. Statistical Analysis

Data analysis was performed using R (R Core Team (2020). R: A language and environment for statistical computing. R Foundation for Statistical Computing, Vienna, Austria. URL https://www.R-project.org/ version 4.0.2, accessed on 22 June 2020). 

After initial assessment of the data for normality using the Shapiro–Wilk test, descriptive statistics were presented with median [25th, 75th quartiles] for variables with non-normal distribution, while mean ± standard deviation (sd) for variables with normal distribution was used. For binary (e.g., presence of AL, need for surgical revision) or categorical (e.g, applied surgery technique, visual appearance of the fluid collection) variables, contingency tables were created. The independence of these variables was assessed with chi^2^- or Fisher’s exact tests, depending on the size of the respective contingency tables (Fisher’s exact test for fourfold tables, otherwise chi^2^-tests). A Bonferroni correction for multiple comparison was applied.

For analysis of the 30-day post-interventional time course of the values of the blood parameters, generalized linear mixed models (GLMM) were carried out. Previously, these data were log-transformed to achieve a normal distribution. Fixed effects were given by the number of days after the intervention and the adjustment for the presence of AL and proof of germs in the drainage fluid. Random intercepts were included by subject ID repeated by days.

Differences between the radiation exposure in the two time periods was assessed with Mann-Whitney tests for independent samples. 

Differences in the horizontal insertion angle at the different vertebral body heights were assessed with a Kruskal-Wallis test followed by pairwise post hoc Wilcoxon tests with Holm correction.

A level of significance of α = 0.05 was used throughout the study.

## 3. Results

### 3.1. Patient Collective

Forty patients (10 women; mean age 63.2 ± 12.6 years) having undergone CT-guided drain placement following colorectal surgery between 2005 and 2020 were included. All patients suffered from adenocarcinomas, which were most frequently located in the rectum (37/40 cases, 92.5%). The most common tumor stage was T3 (55.0%), and 17 of 40 (42.5%) patients had already developed metastases. The organ most frequently affected by metastasis was the liver (*n* = 7, 17.5%). Detailed information on the stage and extension of the disease is provided in Supplementary Table S1.

Approximately two-thirds of patients (26/40, 65.0%) received neoadjuvant therapy. Twenty-three patients (57.5%) were administered neoadjuvant radiochemotherapy. All of them were patients with rectal cancer.

Twenty-eight patients (70.0%) underwent open surgery and 12 patients (30.0%) underwent laparoscopic surgery. In the majority of cases (77.5%), a low anterior rectal resection was performed. In four additional patients (10.0%), this was supplemented by either a sigmoid resection (*n* = 3) or a left hemicolectomy (*n* = 1). Two patients (5.0%) received a hemicolectomy alone. A total of three further operations (7.5%) were performed, comprising the explorative laparotomy and biopsy of a tumor in the pelvis as well as an abdominoperineal rectal resection. In six patients (15.0%), additional organ resections were performed. Some patients underwent multiple resections. The detailed list is shown in Table 1.

Fifteen patients (37.5%) did not receive a stoma, and in most cases (52.5%) an ileostomy was created.

### 3.2. Pre- and Peri-Interventional Analysis

Detailed information on drains and intervention techniques is provided in Table 2. In the last CT examination before the intervention, 22 patients (55.0%) were suspected to have an AL as the cause of the fluid collection. Confirmed AL were present in 25/40 (62.5%) of the patients (mostly by detection of the insufficiency by endoscopy, partly also only during the post-interventional course), including 19/22 (86.3%) patients who showed findings suspicious for AL in the planning CT. The duration between surgery and drain placement varied considerably in the investigated population. The median value was 29 days. In the case of the shortest period, drain placement occurred six days after surgery; the longest interval was 1126 days in a patient with late-stage AL. Six patients were in a septic state at the time of the intervention. In 92.5% of the cases (*n* = 37), one drain was inserted during the intervention, whereas in 7.5% (*n* = 3) patients two drains were applied (mean ± SD: 1.1 ± 0.27 drains per patient). The trocar technique was used in 40 drain placements (93.0%) and the Seldinger technique was used in three drain placements (7.0%). Presacral fluid retention was evident in 39 patients (97.5%). One collection (2.5%) was localized in the precoccygeal region. The smallest collection was 3.7 cm and the largest was 13.8 cm in diameter. On average, all of the lesions had a mean diameter of 6.2 ± 2.3 cm.

CT signs of infection of the fluid collection were seen in 32 cases (80.0%). In 28 of these 32 cases (87.5%), the microbiological evaluation was also positive during the course. 

Twenty-nine collections were punctured via a parasacral approach route (72.5%). Ten (25.0%) used a paracoccygeal and one lesion used an infracoccygeal trajectory (2.5%). The drain was inserted below the piriformis muscle in 32 cases (80.0%), while a transpiriform access was chosen in eight cases (20.0%). The horizontal insertion angle of the drain perpendicular to the ventral margin of the sacrum was (median [25%, 75% quartile]) 55 (48, 67) degrees at the level of the third sacral vertebra, 40 (33.5, 45) degrees at the level of the fourth sacral vertebra, 25 (17, 32) degrees at the level of the fifth sacral vertebra, and 12.5 (9.25, 15.75) degrees at the level of the first coccygeal vertebra. These values differed significantly from each other (*p* < 0.05).

In 37 patients only one drain was placed, while two drains were placed synchronously in three patients. The most commonly used drain diameters were 8 F and 10 F in size (each *n* = 19 (44.2%)), followed by 12 F drains (*n* = 3 (7.0%)). A 7.5 F and a 14 F drain were each used in one case (2.3%).

**Figure 2:**
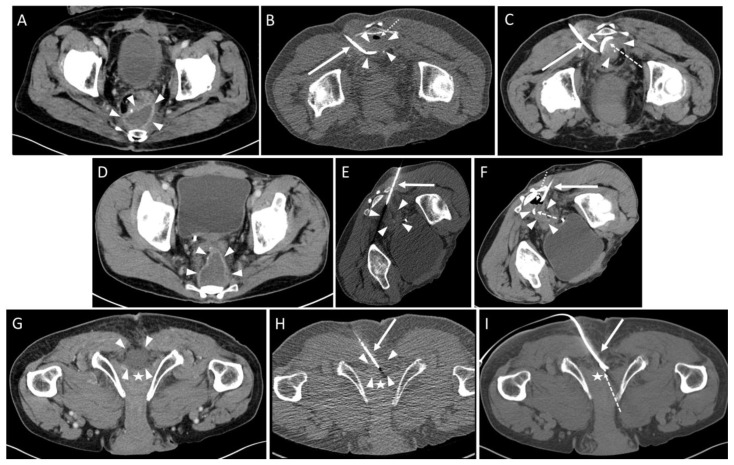
Three cases of pelvic fluid collections after colorectal cancer surgery. Three different access trajectories are depicted: **Upper row:** Transpiriform access path. of a 69 year-old male with a history of rectal carcinoma (T2 tumor stage) after deep anterior rectal resection including total mesorectal excision and the creation of a loop-iliostomy. Partial anastomotic insufficiency with concomitant presacral abscess was observed after two months and initially treated using endoluminal vacuum therapy. Subsequently, a CT fluoroscopy guided drain was inserted after four months (Leukocytes 8.3 × 10^9^/L; CRP: 1.6 mg/dL). (**A**) A preinterventional contrast enhanced CT shows a presacral fluid collection with marked rim enhancement (arrowheads). Loculated small gas collection indicating a fistula and superinfection. (**B**) CT fluoroscopy-guided insertion of a 10 F drain (arrow). Arrowheads: fluid collection; dotted arrow: loculated gas collection. (**C**) Postinterventional control scan. Reduced size of the fluid collection (arrowheads) after aspiration. Arrow: drain; dashed arrow: loop-formation of the distal drain segment. Microbiological analysis revealed infection with Enterococcus faecalis, Haemophilus parainfluenzae, Escherichia coli, Proteus vulgaris, Citrobacter koseri, and Bacteroides uniformis. **Middle row:** Infrapiriform access path. A 51 year-old male with history of hepatic metastatic rectal cancer (T3 tumor stage) and deep anterior rectal resection with Hartmann’s operation after neoadjuvant chemotherapy with FOLFOX4/FOLFIRI schema. Two months after surgery the patient presented with elevated temperature and fever spikes up to 39.5 degrees C (Leukocytes 17.4 × 10^9^/L; CRP: 5.4 mg/dL). A CT fluoroscopy-guided drain was applied. (**D**) A preinterventional contrast enhanced CT showed a presacral fluid collection with marked rim enhancement (arrowheads). (**E**) CT fluoroscopy placement of an 8 F drain (arrow) in semi-prone position. Arrowheads: fluid collection. (**F**) Postoperative control scan. After aspiration of the abscess cavity, a loculated small collection of air could be seen (dotted arrow). The size of the fluid collection (arrowheads) was reduced. A microbiological analysis revealed an infection with Bacteroides fragilis. **Lower row:** Transperineal access path. of a 74 year-old male with history of a deep-seated rectal carcinoma (yT3 tumor stage) after laparoscopic abdominoperineal rectum resection. The patient had fever of up to 39 degrees with recurrent spikes since the 9th postoperative day. Fifteen days after surgery, a CT revealed postoperative fluid retention in the pelvic floor extending to the penile root (Leukocytes 6.8 × 10^9^/L; CRP: 6.9 mg/dL). (**G**) A contrast enhanced planning CT scan in the prone position did not show rim enhancement or air bubbles within the collection (arrowheads). Asterisk: penile root. (**H**) CT fluoroscopy-guided insertion of an 8 F single lumen pigtail drain (arrow) into the perineal region using the Trocar-technique was performed. Arrowheads: fluid collection; asterisk: penile root. (**I**) Complete resolution of the fluid collection after aspiration was seen in the control scan. Arrow: drain; dashed arrow: loop-formation of the distal drain segment; asterisk: penile root. A microbiological analysis revealed infection with Proteus mirabilis.

In 40/43 (93.0%) drain insertions, placement into the fluid collection was technically successful (Figure 2).

**Figure 3:**
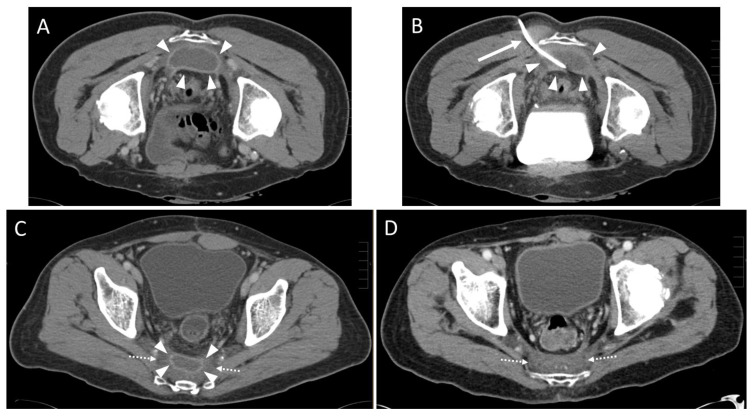
A 59-year-old male with history of rectal cancer (T3 tumor stage) after deep anterior rectal resection including total mesorectal excision and creation of a loop-iliostomy. A control CT scan after 9 weeks revealed a presacral contrast enhancing fluid collection. The patient had no clinical symptoms (Leukocytes: 4.1 × 10^9^/L; CRP: 0.6 mg/dL). (**A**) Preinterventional contrast enhanced CT scan in prone position showing the presacral fluid collection with rim enhancement (arrowheads). (**B**) Insertion of an 8 F drain (arrow) into the abscess formation (arrowheads). Only 20 mL of fluid could be aspirated. A microbiological analysis revealed an encapsulated hematoma infected with peptostreptococcus species. (**C**) Six weeks after the intervention, the fluid accumulation (arrowheads) showed a slight regression in size. Hypodense scar tissue (dotted arrows) had developed. (**D**) After one year the hematoma was completely resolved. Dotted arrows: scar tissue.

**Figure 4:**
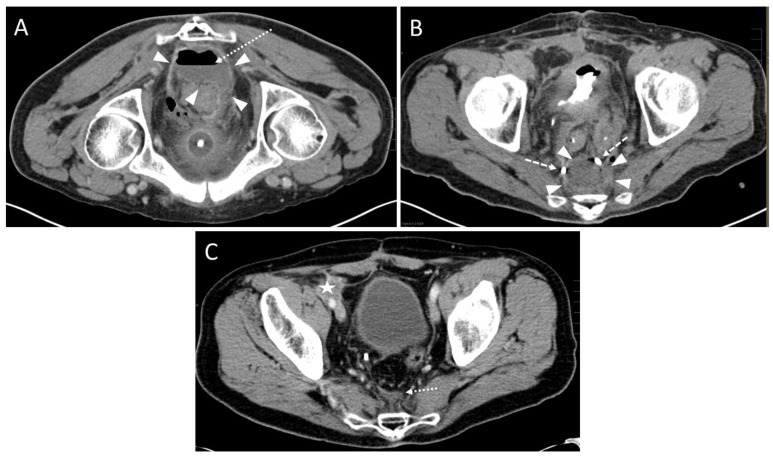
A 67-year-old male with a history of rectal carcinoma (T4 tumor stage) after deep anterior rectal resection including total mesorectal excision and the creation of a loop-iliostomy. In addition, a ureter reimplantation due to tumor infiltration in the bladder and an iliac lymph node dissection was performed. Five days after the operation a small anastomotic insufficiency was diagnosed and treated by flushing. Three weeks later a CT fluoroscopy-guided drain placement was performed due to recurrent fever spikes up to 38.5 degrees. (**A**) A preinterventional scan showed a presacral fluid collection (arrowheads) with air/fluid level (dotted arrow). (**B**) After the CT fluoroscopy-guided insertion of an 8 F drain, purulent fluid could be partially aspirated and a postinterventional control scan showed a moderate reduction of the abscess formation (arrowheads). Dashed arrow: loop-formation of the distal drain segment. (**C**) After one year, the complete resolution of presacral abscess formation was observed. Dotted arrow: small scar tissue. However, a newly appeared soft tissue mass in terms of a peritoneal tumor relapse occurred in the right parailiac region (asterisk).

In one case the drain could not be placed within the abscess cavity due to scar tissue, and the intervention was aborted. In two other cases, each with an infected hematoma (Figure 3) and an abscess formation (Figure 4), only a small amount of fluid could be aspirated. The fluid formations persisted for a longer period of time after the intervention.

Complications occurring during or immediately after the intervention were not observed. Accordingly, the complication rate according to the SIR criteria was 0%. 

CRP at baseline (day of the intervention) was (median [25%, 75% quartile]) 8.2 (5.3; 11.82) mg/dL, Leukocytes were 9.1 (6.5; 13.8) × 10^9^/L and Interleukin-6 was 75.9 (54.1; 99.0) pg/dL. Elevated baseline levels (>0.5 mg/dL) with respect to CRP were observed in 24/24 interventions (100.0%), regarding Leukocytes (>9.8 × 10^9^/L) in 14/30 interventions (47.0%) and with regard to Interleukin-6 (>5.9 pg/dL) in 3/3 interventions (100.0%). In 10 interventions, baseline values were not available.

From 2005 to 2012, 16 interventions were performed with the 16-slice scanner. From 2013 to 2020, a total of 24 procedures were conducted on the two 128-slice scanners. The dose parameter DLP (see Supplementary Figure S2) for the entire interventional procedure was higher during the observation period from 2005 to 2012 (median [25%, 75% quartile]: 735.5 (548.5; 780.75) mGy*cm) than from 2013 to 2020 (544.0 (463.5; 605.0) mGy*cm). The individual components of the DLP for the preinterventional planning scan and for the postinterventional control were also higher in the first observation period than in the second period (2005–2012: planning scan 331.0 (184.5, 370.0) mGy*cm, control scan: 220.5 (136.0, 294.5) mGy*cm vs. 2013–2020: planning scan: 282.0 (246.4; 313.0) mGy*cm; control scan: 194.0 (158.0, 212.0) mGy*cm). These differences were statistically not significant (*p* > 0.143). However, the DLP for the fluoroscopy component was significantly (*p* = 0.033) lower in 2013 to 2020 (47.0 (26.3; 58.5) mGy*cm) than in 2005 to 2012 (85.0 (54.0; 213.0) mGy*cm).

### 3.3. Post-Interventional Analysis

**Figure 5:**
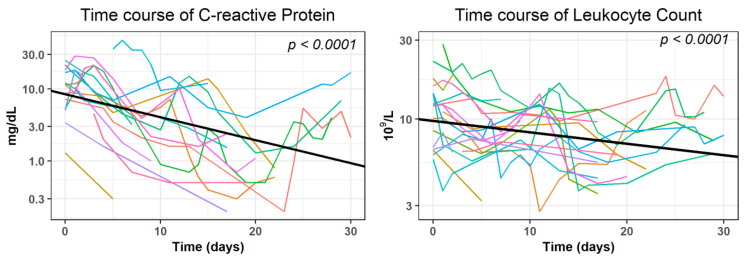
Development of laboratory parameters within 30 days after the intervention in subjects (*n* = 19) with no evidence of further surgical interventions or complications in the patient record.

A statistically significant (*p* < 0.0001) decrease within 30 days after the intervention was observed in the time course of CRP and Leukocytes when analyzed with GLMMs in the subgroup of patients where no evidence of further surgical interventions or complications was given (*n* = 19). Interleukin-6 was excluded, since only 11 measures were documented in this subgroup. The covariate presence of AL and proof of germs in the drainage fluid were statistically not significant (*p* > 0.159). They were excluded from further GLMM analysis (see Supplementary Table S2 for the results of the final regression models). The decrease of the log-transformed average values was as follows: −0.0318 mg/dL for CRP and −0.0069 × 10^9^/L for Leukocytes. (Figure 5).

According to our definition, clinical success (the normalization or decrease of 50% of initially elevated inflammatory parameters) was obtained in 20 out of 24 interventions (83.3%) for CRP after (median [25%, 75% quartile]) 5 (4, 8) days, for Leukocytes in 11 out of 14 interventions (78.6%) after 4 (3, 9) days, and for Interleukin-6 after 5 (3, 6) days in three out of three cases (100.0%).

Supplementary Table S3 shows the patient success rate among different applied surgical procedures. A statistical statement about the distribution of the response rate of CRP and Leukocytes in the resection procedures or about the distribution of Interleukin-6 was not possible due to the small group size. Considering all patients, CRP (82.4%) showed a slightly more frequent normalization than leukocytes (72.7%). However, this difference was not statistically significant (*p* > 0.05).

After drain placement, a total of five patients underwent reoperations that could be attributed to an unfavorable postinterventional outcome: In two patients (5.0%), surgical revisions were performed during the hospital stay at day 9 and 12 after the drain placement due to insufficient fluid collection drainage and persistent inflammatory blood parameters. Three patients (7.5%) with AL (one early type, two late type) could be discharged after drain placement since the clinical symptoms improved temporarily but required surgical revision after 15, 56, and 241 days, respectively. These cases correspond to grade C according to the Rhabari classification. The remaining patients (35/40, 87.5%) were not reoperated on for insufficient drainage and thus corresponded to Rhabari grade B.

Microbiological specimens of wound secretions could be obtained in all except one intervention, and were confirmed to be positive in 33/39 (84.6%) cases. The number of positive results was comparable in interventions with AL (21/25, 84.0%) and in interventions without AL (12/15, 80.0%). The most common strains of detected bacteria were Escherichia, found in 21 patients, and Enterococci, found in 20 patients. The most frequent pathogenic fungus was Candida, which was the underlying germ in four patients. A detailed presentation of the microbiological results is depicted in Supplementary Figure S3.

Comparing infected and non-infected fluid collections, the success rates are presented in Table 3.

The proportion of patients with a significant decrease of laboratory parameter values was higher in the case of an infected fluid collection and highest at 88.9% as far as CRP was concerned.

The visual appearance of the drainage fluid was documented in 32 cases (Supplementary Table S4). The fluid collections appeared significantly (*p* < 0.001) more often to be purulent (16/32, 50.0%). In addition, significantly more germs were positively detected when the drainage fluid was purulent (positive in 15 out of 16 cases (93.8%) vs. negative in 1 out of 16 cases (6.2%); *p* < 0.001). A serous or stool-like aspirate was significantly less frequent (in 2 out of 40 cases (5.0%)). However, the presence of an AL had no significant influence on the visual appearance of the drainage fluid (*p* > 0.05).

Documented average time to drainage removal was (median [25%, 75% quartile]) 6 (3; 12) days. Hospitalization after drain placement was 11.5 (7, 22) days.

## 4. Discussion

We studied the outcome of CT fluoroscopy-guided drainage of deep pelvic fluid collections in patients after colorectal surgery over a period of 16 years. Abdominal fluid collections are a common complication and occur in up to 6% of the cases [33]. In the minority of cases, fluid collections after colorectal surgery may be superinfected due to underlying anastomotic leakages in addition to usual causes for sterile collections such as hematomas, seromas, etc.

Several studies investigated the technical success of percutaneous drain placement for intra-abdominal collections after major abdominal surgery and found a complication rate between 4% and 7% [34,35,36,37,38,39,40,41]. The rate in our study was 7% and was thus in agreement with the aforementioned authors. In the 2010 SIR guidelines, Wallace et al. [42] recommend a threshold of less than 10% for minor or major complications. However, such events did not occur in our study. The drain could not be placed in only one patient (2.5%) due to interposed scar tissue, and the intervention was aborted. A complicated course or iatrogenic damage to the patient could thus be avoided. In two other patients (5.0%), placement was successful, but only minor aspiration was possible. Benoist et al. [43] showed that, among other factors, an abscess diameter of less than 5 cm can be a predictive factor for the failure of a successful placement. The abscess sizes of the two aforementioned patients in our study were 4.8 cm and 5.6 cm, and thus—besides the viscosity of the fluid content—may have contributed to inadequate aspiration.

Our results showed that the further the caudal drain is placed, the steeper the angle of insertion in relation to the anterior margin of the sacrum that is possible in order to achieve technical success. This information may be valuable for the training of inexperienced radiology residents, as the access route can be chosen more optimally in advance in cases of presacral collections. The possible repositioning of the drain can thus be avoided and the procedure can be completed more quickly.

The trocar technique was used in 93.0% of our drain placements. In general, the technique with which the IR is most familiar should be used. However, the trocar technique offers certain advantages over the Seldinger technique. According to Jaffe et al. [44], it is faster to perform, one often does not need an assistant, and it is particularly well suited for larger fluid formations. In addition, it is the recommended technique for endocavitary collections where repeated dilation or initial wire placement may be problematic. Thus, the higher number of cases performed with the trocar technique in our study is in agreement with the recommendations of these authors.

The definition of clinical or therapeutic success of drainage is very heterogeneous in the literature. Some authors suggest only the placement of a drain without the need for a consecutive surgical revision [45], while others aim for a reduction of the size of the collection [46] or the absence of septic complications [47]. We used an approach consisting of clinical and laboratory improvement. Clinical success in our study was defined as no need for surgical revision in the following 30 days after drain placement and by a decrease of laboratory inflammatory parameters (CRP, leukocytes and Interleukin-6) by more than 50% or by normalization within the following 30 days after the intervention. Considering the criterion of an avoided reoperation, the success rate in our collective was 87.5%. To evaluate the criterion of decreasing inflammatory parameters and to take into account the heterogeneity of the patients in our study population, we analyzed a subgroup of patients with a ‘regular course’, i.e., without evidence of further surgical interventions or complications during the observation period, and demonstrated a significant (*p* < 0.0001) decline of CRP and leukocytes in this subcollective. The inflammatory parameter Interleukin-6 revealed the same trend, but could not be statistically assessed due to the small number of cases. For the same reason, it was not possible to make a statistical assessment of the course of the inflammatory parameters when the surgical procedures were taken into account. However, the trend toward a decline in the values was also evident here. The CRP and leukocytes also showed a high success rate when the entire collective was considered, with CRP (82.4%) performing slightly better than leukocytes (72.7%).

Applying our definitions, we found comparable success rates to those of other studies. However, several authors report collectives with partly different localizations and different causes for the fluid accumulation. Theisen et al. [40] reported a success rate of 86% in 174 abdominal collections after major abdominal surgery. Akinci et al. [48] found a clinical success rate of 68% in their study in 255 patients with pelvic abscesses. This was defined as complete healing of the collection without re-intervention, which may explain the somewhat lower success rate. In contrast, Lagana et al. [38] demonstrated a high clinical success rate of 92% in a mixed patient group with 95 abdominal and pelvic collections. Betsch et al. [49] studied 75 patients and found that the etiology of the collection also seems to have a significant influence: The rate of clinical successes in the treatment of abscesses following surgery was 87%, whereas abscesses developing due to other causes (i.e., Crohn’s disease or tuberculosis-associated collections) were treated successfully in 76% of cases. The clinical success rate for postoperative collections after major bowel surgery—and thus corresponding to our collective—was 79%. Cinat et al. [50] also demonstrated that a successful outcome is more likely with abscesses that are postoperative. They reported a success rate of 78% for collections after colorectal surgery. In summary, the clinical success rate in our study is at least comparable to that of the authors mentioned above. Since a reoperation was necessary due to a lack of clinical success in only a few patients, we conclude that the curative effect is clearly more pronounced than the temporizing effect in our collective.

The success rates of CRP and Leukocytes tended to be higher in infected than in noninfected collections (CRP: 88.9% vs. 82.0%; Leukocytes: 83.3% vs. 50.0%). They may therefore be better suited to assess measurable success in the former group. However, it must be taken into account that large and metastasized tumors can increase the total CRP value by autonomous CRP production, and that in some cases the CRP is even a better parameter for the prognosis of the patient than an abdominal infection [51,52,53,54].

Comparing the time intervals of 2005–2012 and 2013–2020, a trend for a reduction of the DLP for the whole intervention as well as for the DLP for the pre- and postinterventional CT scans could be observed. The DLP for the part of the intra-interventional CT scans was significantly lower in the second observation period than in the first one. Kloeckner et al. [32] investigated radiation exposure from various CT-guided interventions and defined reference values. As in our collective, the authors reported that approximately 85% of radiation exposure is based on the pre- and postinterventional CT scan, and only a small proportion occurs during the actual intervention. For abdominal drain placement, the authors recorded a median total DLP of 719 mGy*cm and recommended 942 mGy*cm as a threshold. With a total median DLP of 735.5 mGy*cm in 2005 to 2012 and 544 mGy*cm in the years 2013 to 2020, the DLPs in our study were slightly elevated during the first observation period, and were already far below the recommended reference range mentioned above during the second period. This reduction in dose values is presumably due to various causes. First, there have been fundamental technical developments in CT scanners, including the use of tube current modulation, iterative image reconstruction, detector technology, as well as improvements in CT fluoroscopy technology [14,15,55]. In the second half of the observation period, two interventional 128-sclice scanners equipped with angular beam modulation were used in our department [56], and one of them was additionally equipped with a Stellar detector, minimizing electronic noise and cross-talk in the detector and facilitating a higher signal-to-noise ratio (SNR) despite a reduced dose. Another cause is likely the training effect that occurred as the use of CT fluoroscopy increased over time. Due to the growing learning curve of IRs, a combination of low-milliampere CT fluoroscopy and the quick check technique has been increasingly used [57]. In the quick check technique, fluoroscopy images are acquired repeatedly when the position of the needle or the table is changed, rather than applying continuous fluoroscopic acquisition. This reduces the CT fluoroscopy time, which in turn results in a lower radiation dose for the patient and the IR. For the CT fluoroscopy component, the 16-slice scanner used in the first half of the observation period had a frame rate of eight frames per second and an acquisition matrix of 256 × 256 pixels. The two 128-slice scanners on which the interventions were performed from 2013 to 2020 had a CT fluoroscopy acquisition matrix of 512 × 512 pixels and a rate of 10 frames per second. During both time intervals, the final images presented to the IR on the in-room monitor were upscaled to a 1024 × 1024 pixel matrix. These changes in the technical parameters over time as well as the use of the Stellar detector in the latest CT scanner had no adverse effect on the image quality when using the same standard setting of a tube current-time product of 10 mAs.

In our collective, the most frequently isolated bacteria were Escherichia and Enterococci, and the most frequently isolated fungus was Candida. This corresponds to the usually predominant pathogens in intra-abdominal infections [58,59]. It seems all the more important to establish an initial broad antibiotic therapy in the sense of an early multimodal therapy independent of the type of intervention or suspected focus. The recommendation of Bodmann et al. [59] to consider a coverage of gram-positive bacteria such as enterococci can only be endorsed according to our evaluation. In addition, the results underline that in the case of persistent laboratory chemical and clinical inflammatory constellations, despite broad or test-appropriate antibiotic therapy, candidiasis should be promptly considered and antifungal therapy should be added to the therapy regimen.

Some limitations of this study have to be considered. The definition of the success of the drainage placement turned out to be difficult, which is also shown by the manifold different definitions in the previous literature. In this study, a combination of clinical and laboratory parameters have been deliberately chosen as parameters, as in our opinion this most closely reflects the clinical reality. However, clinical and especially laboratory parameters are influenceable in numerous ways and thus do not always express an actual lack of improvement in the situation of a postoperative collection. Due to the retrospective nature of our study, the patient collective included in this study is very heterogeneous. Through a precise definition of the inclusion and exclusion criteria, an attempt was made to ensure comparability among the individual patients as much as possible. This approach reduced the number of patients that were ultimately included. However, in contrast to other studies in which some very mixed collectives were examined, we have a highly selected patient collective.

## 5. Conclusions

Transgluteal or transperineal CT-guided drainage placement in patients with symptomatic fluid collections in the deep pelvis after colorectal tumor surgery shows a very good technical success rate. The clinical response in terms of decreasing inflammatory parameters as well as necessary reoperations can also be rated as very good. Serious complications did not occur. The development of CT scanner technology in recent years has resulted in a significant decrease in radiation exposure due to the CT fluoroscopy component as well as a decrease in the total radiation dose of the entire procedure. Given a curative or temporizing intention, the method is thus suitable as a safe initial alternative to surgical therapy for a large proportion of patients in the postoperative setting after major colorectal cancer surgery.

## Figures and Tables

**Table 1:** Preoperative pretreatment and surgical therapy.

Variable	*n* (%) ^1^
Neoadjuvant therapy	
Radiochemotherapy	23 (57.5%)
Radiation therapy	2 (5.0%)
Chemotherapy	1 (2.5%)
Type of surgery	
Open	28 (70.0%)
Laparoscopic	12 (30.0%)
Surgery techniques	
Deep anterior rectum resection	31 (77.5%)
Deep anterior rectum resection with sigmoid resection	3 (7.5%)
Deep anterior rectum resection with left hemicolectomy	1 (2.5%)
Hemicolectomy	2 (5.0%)
Other	3 (7.5%)
Additionally resected organs	
Cystectomy	3 (7.5%)
Adnexectomy	2 (5.0%)
Hysterectomy	1 (2.5%)
Prostatectomy	1 (2.5%)
Splenectomy	1 (2.5%)
Type of stoma	
Ileostomy	21 (52.5%)
Colostomy	4 (10.0%)

^1^ *n*: numbers (Percentage).

**Table 2:** Information on intervention situs, drains and technique in the 40 CTD procedures.

Time from surgery to first intervention (days):	29 (13.8, 92.2) (6–1126) ^1^
Max. diameter of the fluid collection (cm)	6.2 ± 2.3 (3.7–13.8) ^2^
CT signs of infection	32 (80.0%) ^3^
CT signs for AL ^4^	22 (55.0%) ^3^
Confirmed AL ^4^	Count
Early	12
Late	13
Predominant location of the fluid collection	Count
presacral	39 (97.5%) ^3^
precoccygeal	1 (2.5%) ^3^
Position	Count
Prone	29
Lateral	11
Drains per intervention	Count
1	37 (92.5%) ^3^
2	3 (7.5%) ^3^
Diameter (French)	Count
7.5	1 (2.3%) 3
8	19 (44.2%) ^3^
10	19 (44.2%) ^3^
12	3 (7.0%) ^3^
14	1 (2.3%) ^3^
Technique	Count
Trocar	40 (93.0%) ^3^
Seldinger	3 (7.0%) ^3^
Approach	
parasacral	29 (72.5%) ^3^
paracoccygeal	10 (25.0%) ^3^
infracoccygeal	1 (2.5%) ^3^
Access path	Count
transpiriform	8 (20.0%) ^3^
infrapiriform	31 (77.5%) ^3^
transperineal	1 (2.5%) ^3^
Aspirated Fluid Volume [mL]	20 (9.8, 50) (3–80) ^1^

^1^: Median [25%, 75% percentile] (range), ^2^: Mean value ± standard deviation (range), ^3^: Numbers (Percentage), ^4^: Anastomotic leakage.

**Table 3:** Distribution of the success rate in terms of decreasing laboratory parameters between infected and non-infected fluid collections.

Fluid Collection Infection Status	C-Reactive Protein			Leukocytes			Interleukin-6		
	Elevated (n)	Success (n, %)	No Success (n, %)	Elevated (n)	Success (n, %)	No Success (n, %)	Elevated (n)	Success (n, %)	No Success (n, %)
Infected	18	16 (88.9)	2 (11.1)	12	10 (83.3)	2 (16.7)	2	2 (100.0)	0 (0.0)
Non-infected	6	4 (82.0)	2 (18.0)	2	1 (50.0)	1 (50.0)	1	1 (100.0)	0 (0.0)
Total	24	20 (83.3)	4 (33.3)	14	11 (78.6)	3 (21.4)	3	3 (83.3)	0 (0.0)

n: number; %: percentage.

## Data Availability

The data presented in this study are available upon reasonable request from the corresponding author.

## Supplementary Materials

The following supporting information can be downloaded at: https://www.mdpi.com/article/10.3390/diagnostics13040711/s1, Table S1: Overview on stage and extension of the disease; Table S2: Parameters of the generalized linear mixed models (GLMM) used in Figure 5; Table S3: Distribution of the success rate in terms of decreasing laboratory parameters among the different applied surgical procedures; Table S4: Visual appearance of the drainage fluid depending on the infection status and presence of anastomotic leakage; Figure S1: Determination of horizontal insertion angle and horizontal insertion height of the drain; Figure S2: Boxplots of the median radiation dose during the observation periods; Figure S3: Microbiological results.

## References

[1] Baidoun F., Elshiwy K., Elkeraie Y., Merjaneh Z., Khoudari G., Sarmini M.T., Gad M., Al-Husseini M., Saad A. (2021). Colorectal Cancer Epidemiology: Recent Trends and Impact on Outcomes. Curr. Drug Targets.

[2] Brenner H., Kloor M., Pox C.P. (2014). Colorectal cancer. Lancet.

[3] Sinicrope F.A. (2022). Increasing Incidence of Early-Onset Colorectal Cancer. N. Engl. J. Med..

[4] Mármol I., Sánchez-de-Diego C., Pradilla Dieste A., Cerrada E., Rodriguez Yoldi M.J. (2017). Colorectal Carcinoma: A General Overview and Future Perspectives in Colorectal Cancer. Int. J. Mol. Sci..

[5] Haraldsdottir S., Einarsdottir H.M., Smaradottir A., Gunnlaugsson A., Halfdanarson T.R. (2014). Colorectal cancer—Review. Laeknabladid.

[6] Men S., Akhan O., Koroglu M. (2002). Percutaneous drainage of abdominal abcess. Eur. J. Radiol..

[7] Floodeen H., Hallbook O., Rutegard J., Sjodahl R., Matthiessen P. (2013). Early and late symptomatic anastomotic leakage following low anterior resection of the rectum for cancer: Are they different entities?. Colorectal. Dis..

[8] Gessler B., Eriksson O., Angenete E. (2017). Diagnosis, treatment, and consequences of anastomotic leakage in colorectal surgery. Int. J. Colorectal. Dis..

[9] Hirst N.A., Tiernan J.P., Millner P.A., Jayne D.G. (2014). Systematic review of methods to predict and detect anastomotic leakage in colorectal surgery. Colorectal. Dis..

[10] Rahbari N.N., Weitz J., Hohenberger W., Heald R.J., Moran B., Ulrich A., Holm T., Wong W.D., Tiret E., Moriya Y. (2010). Definition and grading of anastomotic leakage following anterior resection of the rectum: A proposal by the International Study Group of Rectal Cancer. Surgery.

[11] Dariushnia S.R., Mitchell J.W., Chaudry G., Hogan M.J. (2020). Society of Interventional Radiology Quality Improvement Standards for Image-Guided Percutaneous Drainage and Aspiration of Abscesses and Fluid Collections. J. Vasc. Interv. Radiol..

[12] vanSonnenberg E., Mueller P.R., Ferrucci J.T. (1984). Percutaneous drainage of 250 abdominal abscesses and fluid collections. Part I: Results, failures, and complications. Radiology.

[13] Sheafor D.H., Paulson E.K., Simmons C.M., DeLong D.M., Nelson R.C. (1998). Abdominal percutaneous interventional procedures: Comparison of CT and US guidance. Radiology.

[14] Carlson S.K., Bender C.E., Classic K.L., Zink F.E., Quam J.P., Ward E.M., Oberg A.L. (2001). Benefits and safety of CT fluoroscopy in interventional radiologic procedures. Radiology.

[15] Paprottka P.M., Helmberger T., Reiser M.F., Trumm C.G. (2013). Computed tomography guidance: Fluoroscopy and more. Radiologe.

[16] Schweiger G.D., Yip V.Y., Brown B.P. (2000). CT fluoroscopic guidance for percutaneous needle placement into abdominopelvic lesions with difficult access routes. Abdom. Imaging.

[17] vanSonnenberg E., Wing V.W., Casola G., Coons H.G., Nakamoto S.K., Mueller P.R., Ferrucci J.T., Halasz N.A., Simeone J.F. (1984). Temporizing effect of percutaneous drainage of complicated abscesses in critically ill patients. AJR Am. J. Roentgenol..

[18] Harisinghani M.G., Gervais D.A., Maher M.M., Cho C.H., Hahn P.F., Varghese J., Mueller P.R. (2003). Transgluteal approach for percutaneous drainage of deep pelvic abscesses: 154 cases. Radiology.

[19] Longo W.E., Milsom J.W., Lavery I.C., Church J.C., Oakley J.R., Fazio V.W. (1993). Pelvic abscess after colon and rectal surgery—What is optimal management?. Dis. Colon Rectum.

[20] Ren H.J., Zhang J.P., Tian R.X., Wang G.F., Gu G.S., Hong Z.W., Wu L., Zheng T., Zhang H.Z., Ren J.A. (2020). Analysis of the effect of transgluteal percutaneous drainage in the treatment of deep pelvic abscess. Zhonghua Wei Chang Wai Ke Za Zhi.

[21] Zhao N., Li Q., Cui J., Yang Z., Peng T. (2018). CT-guided special approaches of drainage for intraabdominal and pelvic abscesses: One single center’s experience and review of literature. Medicine.

[22] de Kok B.M., Marinelli A., Puylaert J., Cobben L.P.J. (2019). Image-guided posterior transperineal drainage for presacral abscess: An analysis of 21 patients. Diagn. Interv. Imaging.

[23] Peng T., Dong L., Zhu Z., Cui J., Li Q., Li X., Wu H., Wang C., Yang Z. (2016). CT-guided Drainage of Deep Pelvic Abscesses via a Percutaneous Presacral Space Approach: A Clinical Report and Review of the Literature. Acad. Radiol..

[24] Golfieri R., Cappelli A. (2007). Computed tomography-guided percutaneous abscess drainage in coloproctology: Review of the literature. Technol. Coloproctol..

[25] Ballard D.H., Gates M.C., Hamidian Jahromi A., Harper D.V., Do D.V., D’Agostino H.B. (2019). Transrectal and transvaginal catheter drainages and aspirations for management of pelvic fluid collections: Technique, technical success rates, and outcomes in 150 patients. Abdom. Radiol..

[26] Ouyang B.W., Liu T.W., Fu Z.L., Li Y., Zhang B. (2021). Endoscopic ultrasound-guided pelvic abscess drainage: A report of 2 cases and literature review. Z. Gastroenterol..

[27] Poincloux L., Caillol F., Allimant C., Bories E., Pesenti C., Mulliez A., Faure F., Rouquette O., Dapoigny M., Abergel A. (2017). Long-term outcome of endoscopic ultrasound-guided pelvic abscess drainage: A two-center series. Endoscopy.

[28] Young A.S., Shyn P.B., Johnson O.W., Sainani N.I., Nawfel R.D., Silverman S.G. (2017). Bending percutaneous drainage catheters to facilitate CT-guided insertion using curved trocar technique. Abdom. Radiol..

[29] Gupta S., Wallace M.J., Cardella J.F., Kundu S., Miller D.L., Rose S.C. (2010). Quality improvement guidelines for percutaneous needle biopsy. J. Vasc. Interv. Radiol..

[30] vanSonnenberg E., Wittich G.R., Goodacre B.W., Casola G., D’Agostino H.B. (2001). Percutaneous abscess drainage: Update. World J. Surg..

[31] Filippiadis D.K., Binkert C., Pellerin O., Hoffmann R.T., Krajina A., Pereira P.L. (2017). Cirse Quality Assurance Document and Standards for Classification of Complications: The Cirse Classification System. Cardiovasc. Intervent. Radiol..

[32] Kloeckner R., dos Santos D.P., Schneider J., Kara L., Dueber C., Pitton M.B. (2013). Radiation exposure in CT-guided interventions. Eur. J. Radiol..

[33] Sarkissian H., Hyman N., Osler T. (2013). Postoperative fluid collections after colon resection: The utility of clinical assessment. Am. J. Surg..

[34] Dattola A., Alberti A., Giannetto G., Di Marco D., Basile G. (1999). Echo-guided percutaneous drainage of abscesses and abdominal fluid collections. Ann. Ital. Chir..

[35] Jansen M., Truong S., Riesener K.P., Sparenberg P., Schumpelick V. (1999). Results of sonographically guided percutaneous catheter drainage of intra-abdominal abscesses in surgery. Chirurg.

[36] Kim Y.J., Han J.K., Lee J.M., Kim S.H., Lee K.H., Park S.H., An S.K., Lee J.Y., Choi B.I. (2006). Percutaneous drainage of postoperative abdominal abscess with limited accessibility: Preexisting surgical drains as alternative access route. Radiology.

[37] Kumar R.R., Kim J.T., Haukoos J.S., Macias L.H., Dixon M.R., Stamos M.J., Konyalian V.R. (2006). Factors affecting the successful management of intra-abdominal abscesses with antibiotics and the need for percutaneous drainage. Dis. Colon Rectum.

[38] Laganà D., Carrafiello G., Mangini M., Ianniello A., Giorgianni A., Nicotera P., Fontana F., Dionigi G., Fugazzola C. (2008). Image-guided percutaneous treatment of abdominal-pelvic abscesses: A 5-year experience. Radiol. Med..

[39] Röthlin M.A., Schöb O., Klotz H., Candinas D., Largiadèr F. (1998). Percutaneous drainage of abdominal abscesses: Are large-bore catheters necessary?. Eur. J. Surg..

[40] Theisen J., Bartels H., Weiss W., Berger H., Stein H.J., Siewert J.R. (2005). Current concepts of percutaneous abscess drainage in postoperative retention. J. Gastrointest. Surg..

[41] Voros D., Gouliamos A., Kotoulas G., Kouloheri D., Saloum G., Kalovidouris A. (1996). Percutaneous drainage of intra-abdominal abscesses using large lumen tubes under computed tomographic control. Eur. J. Surg..

[42] Wallace M.J., Chin K.W., Fletcher T.B., Bakal C.W., Cardella J.F., Grassi C.J., Grizzard J.D., Kaye A.D., Kushner D.C., Larson P.A. (2010). Quality improvement guidelines for percutaneous drainage/aspiration of abscess and fluid collections. J. Vasc. Interv. Radiol..

[43] Benoist S., Panis Y., Pannegeon V., Soyer P., Watrin T., Boudiaf M., Valleur P. (2002). Can failure of percutaneous drainage of postoperative abdominal abscesses be predicted?. Am. J. Surg..

[44] Jaffe T.A., Nelson R.C. (2016). Image-guided percutaneous drainage: A review. Abdom. Radiol..

[45] Kirat H.T., Remzi F.H., Shen B., Kiran R.P. (2011). Pelvic abscess associated with anastomotic leak in patients with ileal pouch-anal anastomosis (IPAA): Transanastomotic or CT-guided drainage?. Int. J. Colorectal. Dis..

[46] El-Hussuna A., Karer M.L.M., Uldall Nielsen N.N., Mujukian A., Fleshner P.R., Iesalnieks I., Horesh N., Kopylov U., Jacoby H., Al-Qaisi H.M. (2021). Postoperative complications and waiting time for surgical intervention after radiologically guided drainage of intra-abdominal abscess in patients with Crohn’s disease. BJS Open.

[47] Brusciano L., Maffettone V., Napolitano V., Izzo G., Rossetti G., Izzo D., Russo F., Russo G., del Genio G., del Genio A. (2004). Management of colorectal emergencies: Percutaneous abscess drainage. Ann. Ital. Chir..

[48] Akinci D., Ergun O., Topel C., Ciftci T., Akhan O. (2018). Pelvic abscess drainage: Outcome with factors affecting the clinical success. Diagn. Interv. Radiol..

[49] Betsch A., Wiskirchen J., Trubenbach J., Manncke K.H., Belka C., Claussen C.D., Duda S.H. (2002). CT-guided percutaneous drainage of intra-abdominal abscesses: APACHE III score stratification of 1-year results. Acute Physiology, Age, Chronic Health Evaluation. Eur. Radiol..

[50] Cinat M.E., Wilson S.E., Din A.M. (2002). Determinants for successful percutaneous image-guided drainage of intra-abdominal abscess. Arch. Surg..

[51] Kim D.K., Oh S.Y., Kwon H.C., Lee S., Kwon K.A., Kim B.G., Kim S.G., Kim S.H., Jang J.S., Kim M.C. (2009). Clinical significances of preoperative serum interleukin-6 and C-reactive protein level in operable gastric cancer. BMC Cancer.

[52] Kurokawa Y., Yamashita K., Kawabata R., Fujita J., Imamura H., Takeno A., Takahashi T., Yamasaki M., Eguchi H., Doki Y. (2020). Prognostic value of postoperative C-reactive protein elevation versus complication occurrence: A multicenter validation study. Gastric. Cancer.

[53] Mahmoud F.A., Rivera N.I. (2002). The role of C-reactive protein as a prognostic indicator in advanced cancer. Curr. Oncol. Rep..

[54] Nozoe T., Matsumata T., Kitamura M., Sugimachi K. (1998). Significance of preoperative elevation of serum C-reactive protein as an indicator for prognosis in colorectal cancer. Am. J. Surg..

[55] Grosser O.S., Wybranski C., Kupitz D., Powerski M., Mohnike K., Pech M., Amthauer H., Ricke J. (2017). Improvement of image quality and dose management in CT fluoroscopy by iterative 3D image reconstruction. Eur. Radiol..

[56] Hohl C., Suess C., Wildberger J.E., Honnef D., Das M., Mühlenbruch G., Schaller A., Günther R.W., Mahnken A.H. (2008). Dose reduction during CT fluoroscopy: Phantom study of angular beam modulation. Radiology.

[57] Rathmann N., Haeusler U., Diezler P., Weiss C., Kostrzewa M., Sadick M., Schoenberg S.O., Diehl S.J. (2015). Evaluation of radiation exposure of medical staff during CT-guided interventions. J. Am. Coll. Radiol..

[58] Blot S., De Waele J.J. (2005). Critical issues in the clinical management of complicated intra-abdominal infections. Drugs.

[59] Bodmann K.F. (2010). Complicated intra-abdominal infections: Pathogens, resistance. Recommendations of the Infectliga on antbiotic therapy. Chirurg.

