# Supplementary material for: Low-Dose CT Fluoroscopy-Guided Drainage of Deep Pelvic Fluid Collections after Colorectal Cancer Surgery: Technical Success, Clinical Outcome and Safety in 40 Patients
Source: Diagnostics (Basel). 2023 Feb 13;13(4):711. doi: 10.3390/diagnostics13040711 (PMC9955776; doi:10.3390/diagnostics13040711)
Supplement: Supplementary file 1 [file diagnostics-13-00711-s001.zip › diagnostics-2193030-supplementary/supplementary_files/supplementary_figures.docx]

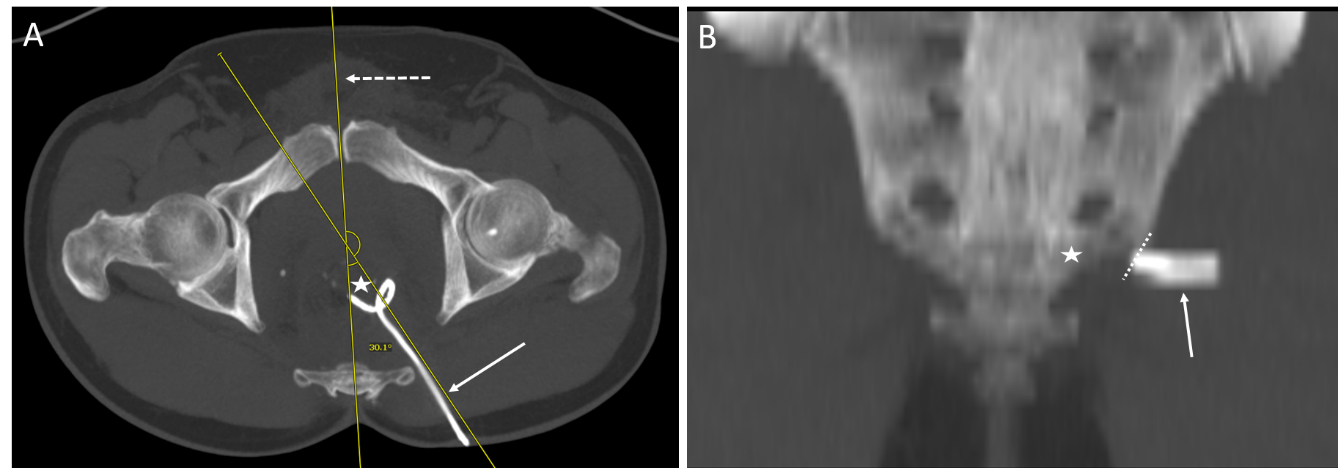


**Supplementary Figure S1:** Determination of horizontal insertion angle and horizontal insertion height of the drain. Determination of angle and insertion height of the drainage system: A) Angle: the axial Maximum-Intensity Projection (MIP; 25 mm slice thickness) showing the longest course of the drain in the puncture channel was selected. A line between the symphyseal joint and the center of the sacrum was drawn. This corresponds to a perpendicular line to the anterior margin of the sacrum (dashed arrow). A second line was drawn parallel to the drain insertion channel (arrow). The acute angle between both lines defines the insertion angle (asterisk, here 30.1 degrees). B) Height: on a coronary reconstruction of the MIP the vertebral body (star, here fourth sacral vertebrae) where the drainage (arrow) crosses the soft tissue-bone boundary (dotted line) was determined.


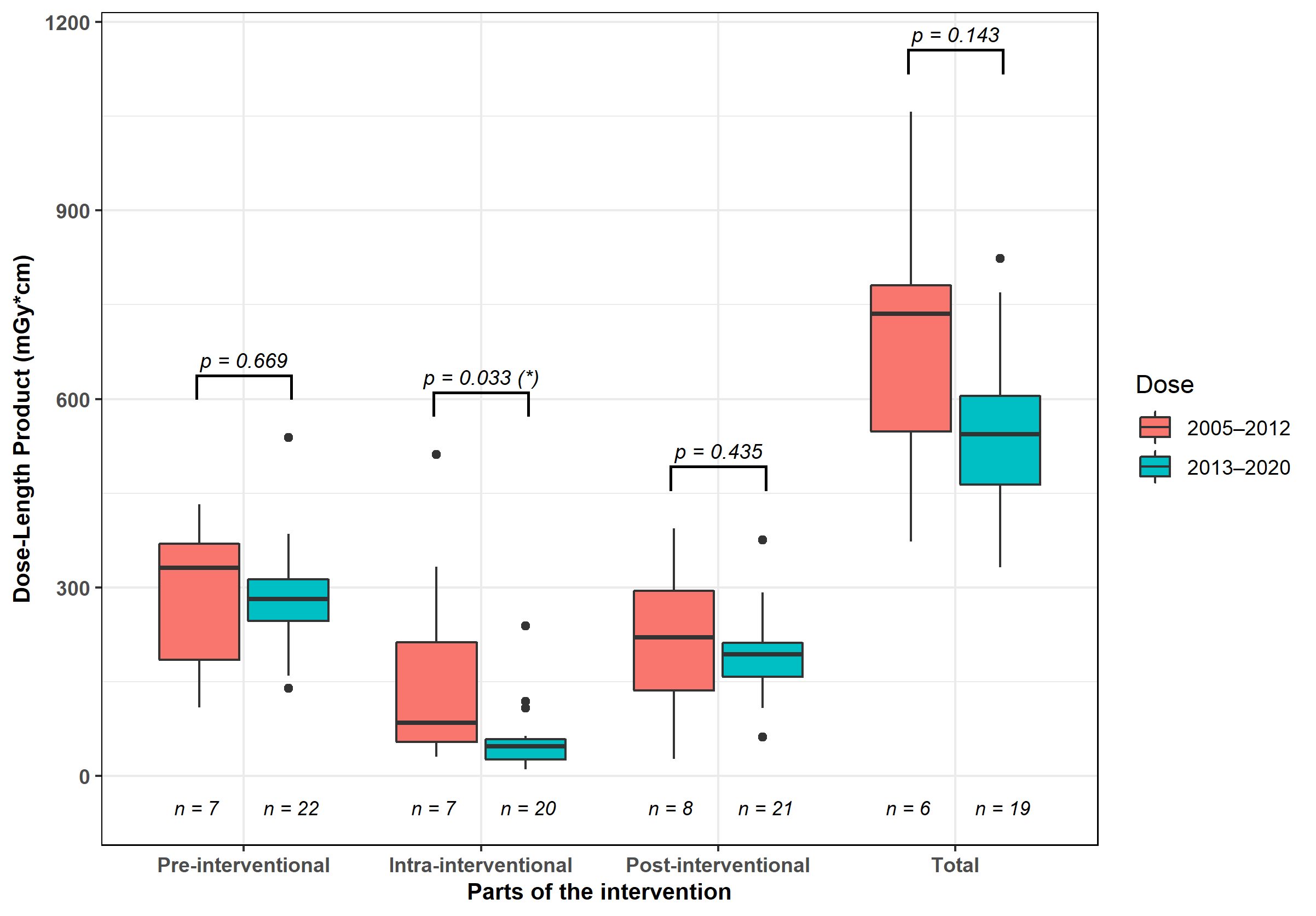


**Supplementary Figure S2:** Boxplots of the median radiation dose during the observation periods. Boxplots of the median radiation dose between the time intervals of the years 2005-2012 and years 2013-2020 for the parts of the interventional CT scan and for the whole procedure. (*): indicates statistically significant difference.


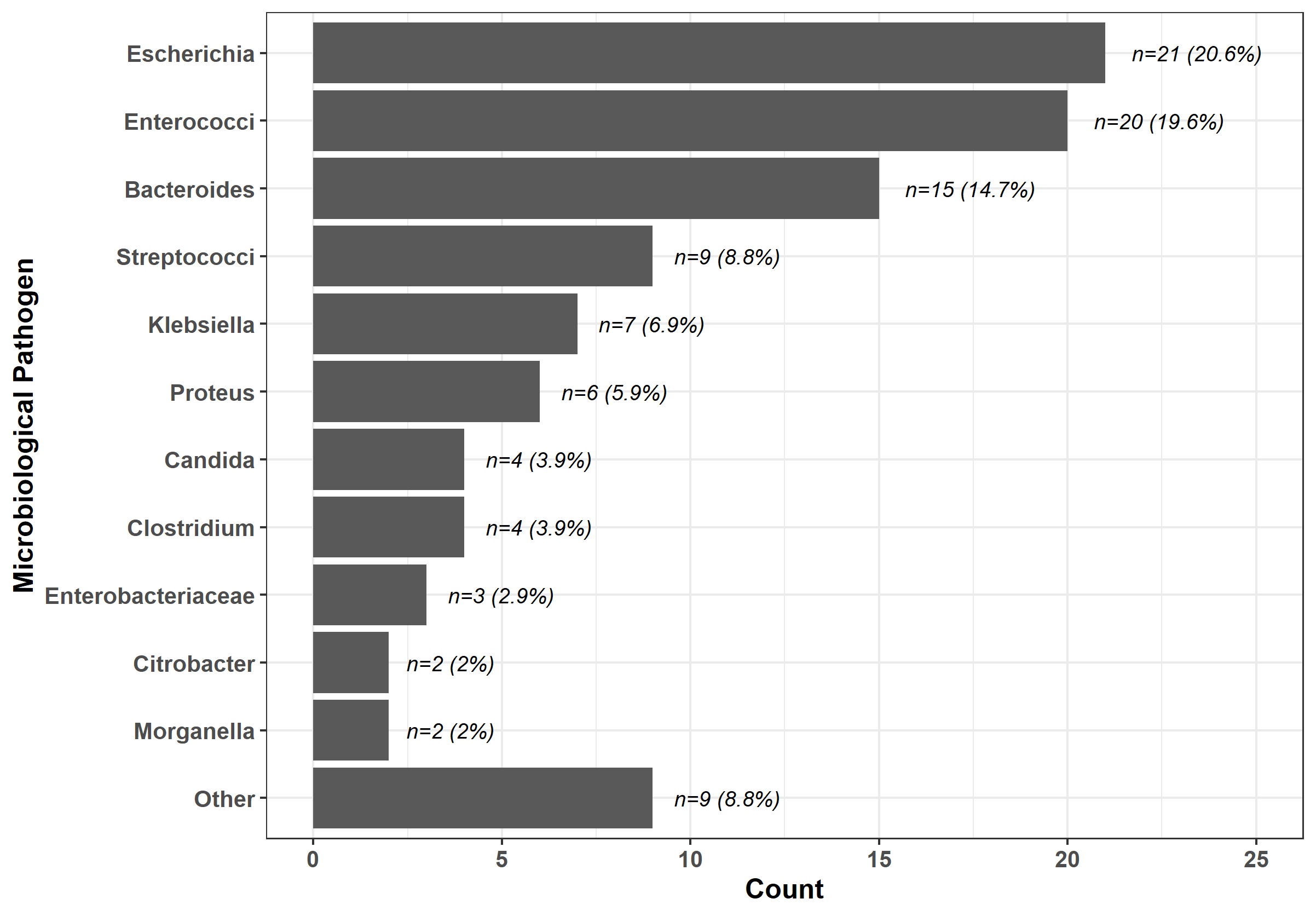


**Supplementary** **Figure S3:** Microbiological results.
