# Supplementary material for: Low-Dose CT Fluoroscopy-Guided Drainage of Deep Pelvic Fluid Collections after Colorectal Cancer Surgery: Technical Success, Clinical Outcome and Safety in 40 Patients
Source: Diagnostics (Basel). 2023 Feb 13;13(4):711. doi: 10.3390/diagnostics13040711 (PMC9955776; doi:10.3390/diagnostics13040711)
Supplement: Supplementary file 1 [file diagnostics-13-00711-s001.zip › diagnostics-2193030-supplementary/supplementary_files/supplementary_tables.docx]

**Supplementary Table S1:** Overview on stage and extension of the disease.

| **Variable** | **n (%) ^1^** |
| --- | --- |
| Tumor location |  |
| Rectum | 37 (92.5%) |
| Sigmoid | 2 (5.0%) |
| Rectosigmoid junction | 1 (2.5%) |
|  |  |
| Tumor stage |  |
| T0 | 2 (5.0%) |
| T1 | 2 (5.0%) |
| T2 | 7 (17.5%) |
| T3 | 22 (55.0%) |
| T4 | 7 (17.5%) |
|  |  |
| Presence of metastases |  |
| Peritoneal | 2 (5.0%) |
| Hepatic | 7 (17.5%) |
| Pulmonary | 2 (5.0%) |
| Other locations | 6 (15.0%) |

^1)^ n: numbers (Percentage).

**Supplementary** **Table S2:** Parameters of the generalized linear mixed models (GLMM) used in Figure 5.

|  | **C-reactive Protein** | | | **Leukocyte Count** | | |
| --- | --- | --- | --- | --- | --- | --- |
| ***Predictors*** | ***Estimates*** | ***CI*** | ***p*** | ***Estimates*** | ***CI*** | ***p*** |
| (Intercept) | 0.93 | 0.72 – 1.13 | **<0.001** | 0.99 | 0.91 – 1.07 | **<0.001** |
| Time (days) | -0.03 | -0.04 – -0.02 | **<0.001** | -0.01 | -0.01 – -0.00 | **<0.001** |
| **Random Effects** | | | | | | |
| σ^2^ | 0.14 | | | 0.02 | | |
| τ_00_ | 0.14 _Subject ID_ | | | 0.02 _Subject ID_ | | |
| ICC | 0.50 | | | 0.57 | | |
| N | 18 _Subject ID_ | | | 19 _Subject ID_ | | |
| Observations | 134 | | | 158 | | |
| Marginal R^2^ / Conditional R^2^ | 0.213 / 0.604 | | | 0.081 / 0.606 | | |

CI: Confidence Interval; R^2^: Coefficient of Determination; σ^2^: distribution-specific variance; τ00: between-subject-variance; ICC: intraclass correlation coefficient, N: number of subjects.

**Supplementary** **Table S3:** Distribution of the success rate in terms of decreasing laboratory parameters among the different applied surgical procedures.

|  |  | **C-reactive Protein** | | |  | **Leukocytes** | | |  | **Interleukin-6** | | |
| --- | --- | --- | --- | --- | --- | --- | --- | --- | --- | --- | --- | --- |
| **Operation technique** |  | Elevated (n) | Success  (n, %) | No Success (n, %) |  | Elevated (n) | Success (n, %) | No Success (n, %) |  | Elevated (n) | Success (n, %) | No Success (n, %) |
| Deep anterior rectum resection |  | 17 | 14 (82.4) | 3 (17.6) |  | 11 | 8 (72.7) | 3 (27.3) |  | 2 | 2 (100.0) | 0 (0.0) |
| Deep anterior rectum resection with sigmoid resection |  | 3 | 2 (66.7) | 1 (33.3) |  | 1 | 1 (100.0) | 0 (0.0) |  | 1 | 1 (100.0) | 0 (0.0) |
| Deep anterior rectum resection with left hemicolectomy |  | 0 |  |  |  | 0 |  |  |  | 0 |  |  |
| Hemicolectomy |  | 1 | 1 (100.0) | 0 (0.0) |  | 1 | 1 (100.0) | 0 (0.0) |  | 0 |  |  |
| Other |  | 3 | 3 (100.0) | 0 (0.0) |  | 1 | 1 (100.0) | 0 (0.0) |  | 0 |  |  |
| Total |  | 24 | 20 (83.3) | 4 (16.7%) |  | 14 | 11 (78.6) | 3 (21.4) |  | 3 | 3 (100.0) | 0 |

n: number; %: percentage.

**Supplementary** **Table S4:** Visual appearance of the drainage fluid depending on the infection status and presence of anastomotic leakage.

| **Visual appearance ^1^** | |  |  | **Proof of germs ^2^** | |
| --- | --- | --- | --- | --- | --- |
|  |  |  | **AL** | *Positive* | *Negative* |
| Bloody | 12 (37.4%) |  | *Present* | 5 (41.7%) | 2 (16.7%) |
|  |  |  | *Not present* | 4 (33.3%) | 1 (8.3%) |
| Purulent | **16 (50.0%)** |  | *Present* | **11 (68.8 %)** | 1 (6.2 %) |
|  |  |  | *Not present* | **4 (25.0 %)** |  |
| Stool-like | **2 (6.3%)** |  | *Present* | 2 (100.0 %) |  |
|  |  |  | *Not present* |  |  |
| Serous | **2 (6.3%)** |  | *Present* | 1 (50.0 %) | 1 (50.0 %) |
|  |  |  | *Not present* |  |  |

^1^: Numbers (Percentage). The group includes all cases where the visual appearance of the drainage fluid was documented (n=32). Percentages refer to the column values. ^2^: Numbers (Percentage). Subgroup in which germ detection and testing for the presence of a anastomosis leakage were performed (n=32). Percentages refer to the values of the corresponding 2x2 contingency table. Values in bold indicates significant result in Chi^2^-test. AL: anastomotic leakage.
